# Interactions Between Estrogen- and Ah-Receptor Signalling Pathways in Primary Culture of Salmon Hepatocytes Exposed to Nonylphenol and 3,3',4,4'-Tetrachlorobiphenyl (Congener 77)

**DOI:** 10.1186/1476-5926-6-2

**Published:** 2007-04-13

**Authors:** Anne S Mortensen, Augustine Arukwe

**Affiliations:** 1 Department of Biology, Norwegian University of science and Technology (NTNU), Høgskoleringen 5, 7491 Trondheim, Norway

## Abstract

**Background:**

The estrogenic and xenobiotic biotransformation gene expressions are receptor-mediated processes that are ligand structure-dependent interactions with estrogen-receptor (ER) and aryl hydrocarbon receptor (AhR), probably involving all subtypes and other co-factors. The anti-estrogenic activities of AhR agonists have been reported. In teleost fish, exposure to AhR agonists has been associated with reduced Vtg synthesis or impaired gonadal development in both *in vivo*- and *in vitro *studies. Inhibitory AhR and ER cross-talk have also been demonstrated in breast cancer cells, rodent uterus and mammary tumors. Previous studies have shown that AhR-agonists potentiate xenoestrogen-induced responses in fish *in vivo *system. Recently, several studies have shown that AhR-agonists directly activate ERα and induce estrogenic responses in mammalian *in vitro *systems. In this study, two separate experiments were performed to study the molecular interactions between ER and AhR signalling pathways using different concentration of PCB-77 (an AhR-agonist) and time factor, respectively. Firstly, primary Atlantic salmon hepatocytes were exposed to nonylphenol (NP: 5 μM – an ER agonist) singly or in combination with 0.001, 0.01 and 1 μM PCB-77 and sampled at 48 h post-exposure. Secondly, hepatocytes were exposed to NP (5 μM) or PCB-77 (1 μM) singly or in combination for 12, 24, 48 and 72 h. Samples were analyzed using a validated real-time PCR for genes in the ER pathway or known to be NP-responsive and AhR pathway or known to be PCB-77 responsive.

**Results:**

Our data showed a reciprocal inhibitory interaction between NP and PCB-77. PCB-77 produced anti-NP-mediated effect by decreasing the mRNA expression of ER-responsive genes. NP produced anti-AhR mediated effect or as inhibitor of AhRα, AhRR, ARNT, CYP1A1 and UDPGT expression. A novel aspect of the present study is that low (0.001 μM) and medium (0.01 μM) PCB-77 concentrations increased ERα mRNA expression above control and NP exposed levels, and at 12 h post-exposure, PCB-77 exposure alone produced significant elevation of ERα, ERβ and *Zr*-protein expressions above control levels.

**Conclusion:**

The findings in the present study demonstrate a complex mode of ER-AhR interactions that were dependent on time of exposure and concentration of individual chemicals (NP and PCB-77). This complex mode of interaction is further supported by the effect of PCB-77 on ERα and ERβ (shown as increase in transcription) with no concurrent activation of Vtg (but *Zr*-protein) response. These complex interactions between two different classes of ligand-activated receptors provide novel mechanistic insights on signalling pathways. Therefore, the degree of simultaneous interactions between the ER and AhR gene transcripts demonstrated in this study supports the concept of cross-talk between these signalling pathways.

## Background

Halogenated organic contaminants such as 2,3,7,8-tetrachlorodibenzo-p-dioxin (TCDD), polychlorinated biphenyls (PCBs), and polycyclic aromatic hydrocarbons (PAHs) are notorious environmental pollutants that cause acute and chronic toxicity [1]. Several of these compounds including planar PCBs, exert their biological effects through the aryl hydrocarbon receptor (AhR or Ah-receptor). The AhR is a ligand activated transcription factor that regulates the activation of several genes encoding phase I and II biotransformation enzymes [2]. The AhR belongs to the family of basic helix-loop-helix (BHLH)/Per-ARNT-Sim (PAS) proteins that are characterized by two conserved domains, the N-terminal bHLH and the PAS domain [2,3]. Cytochrome (CYP) P450 enzymes (CYP1A1, 1A2, 1B1) are involved in the metabolism of a wide variety of structurally different chemicals that include many drugs and xenobiotics, through the AhR [2,3]. For example, the molecular mechanism of CYP1A activation has been extensively studied. Prior to ligand binding, the cytosolic form of the AhR is associated with a chaperone complex consisting of heat shock protein 90 (hsp90) and several other co-chaperones [2,3]. Upon ligand binding, the AhR is released from the hsp90 complex and translocated into the nucleus where it dimerizes with a structurally related protein, the AhR nuclear translocator (ARNT). The AHR/ARNT complex binds with high affinity to specific DNA sequences known as dioxin or xenobiotic response elements (DREs or XREs) located in the regulatory regions of target genes leading to their activation and expression. In addition to CYP enzymes, phase-II enzymes such as uridine-diphosphate glucuronosyltransferase (UDPGT) are now known to be inducible through the AhR [2,3] and these responses are putatively controlled through the AhR repressor (AhRR: [2]). Thus, AhR controls a battery of genes involved in the biotransformation of xenobiotics [2,3].

In oviparous animals, accumulation of yolk materials into oocytes during oogenesis and their mobilization during embryogenesis are key processes for successful reproduction [4,5]. Similarly, the envelope (*zona radiata *or *Zr*) surrounding the animal egg plays significant roles in the reproductive and developmental processes; firstly as an interface between the egg and sperm, and secondly as an interface between the embryo and its environment [4,5]. Vitellogenesis and zonagenesis are estrogen receptor (ER)-mediated estradiol-17β (E2)-induced hepatic synthesis of egg yolk protein (Vtg) and eggshell protein (*Zr*-protein) precursor, respectively, their secretion and transport in blood to the ovary and their uptake into maturing oocytes [4,5]. The ERs (ERα and ERβ) are members of the nuclear receptor (NR) gene superfamily. The ERs bind to estrogen response elements (EREs) and activate transcription in an estrogen concentration-dependent manner [6]. This transcriptional activation requires the recruitment of co-activator complexes [6]. Xenoestrogens, such as nonylphenol (NP) were shown to induce hepatic expression of Vtg and *Zr*-protein genes in immature and male fish [7]. NP predominantly occurs as a degradation product of nonylphenol ethoxylate (NPE), found in many types of products, including detergents, plastics, emulsifiers, pesticides, and industrial and domestic cleaning products.

There are many potential xenobiotics and xenoestrogens in aquatic systems (*e.g.*, pharmaceuticals, pesticides, surfactants and personal care products). Thus, in the environment, chemical interactions may have profound consequences since organisms, including fish, are exposed to complex mixtures of environmental pollutants [8]. These complex interactions have only recently become the focus of systematic investigations both in laboratory and elsewhere [8,9]. The anti-estrogenic activities of AhR agonists have been reported [10]. In fish, exposure to AhR agonists has been associated with reduced Vtg synthesis or impaired gonad development in both *in vivo*- and *in vitro *studies [11,9,12]. Inhibitory AhR-ER cross-talk has been demonstrated in breast cancer cells, rodent uterus and mammary tumors [13].

The relative importance of the influence of contaminants on biological systems is not well-understood or quantified mechanistically in complex chemical mixtures. PCB-77 is a documented AhR agonist with anti-estrogenic activity and was previously shown to increase and decrease (depending on dose ratios, season and sequential order of administration) NP-induced responses in Atlantic salmon (*Salmo salar*) *in vivo *system [11]. In toxicological sciences, almost without exception, gene expression is altered as either a direct or indirect result of toxicant exposure. Depending upon the severity and duration of the toxicant exposure, genomic analysis may be short-term toxicological responses leading to impacts on survival and reproduction (parental and offspring fitness). Therefore, gene expression profiling has become a powerful tool in molecular biology with potential to reveal genetic signatures in organisms that can be used to predict toxicity of these compounds [14]. Therefore, the present study was designed with the objective of investigating the concentration- and time-dependency of interactions (cross-talk) between the ER and AhR signalling pathways using molecular approaches. In addition, we wanted to establish in parallel, the time-dependency of the potential bi-directional cross-talk between these two signalling pathways.

## Results

Based on previous studies in our laboratory, we selected 5 genes (ERα, ERβ, Vtg, *Zr*-proteins and vigilin) belonging to the ER-pathway or known to be ER-responsive and 7 genes (AhRα, AhRβ, AhRR, ARNT, CYP1A1, UDPGT and a proteasome subunit) in the AhR-pathway or known to be AhR-responsive for quantitative analysis using real-time PCR with gene specific primers. Several subtypes of ARNT and UDPGT have been characterized in fish and the primer sequences used in the real-time PCR assays were designed based on conserved regions of these genes.

### Concentration-dependent expression of ER-responsive genes

**Figure 1:**
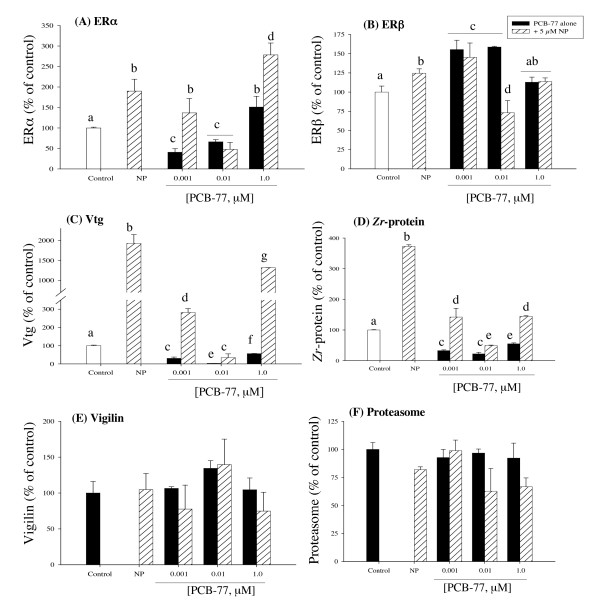
**Expression of ERα (A), ERβ (B), Vtg (C), *Zr*-protein (D), vigilin (E) and 20S proteasome subunit (F) mRNA in primary culture of salmon hepatocytes exposed for 48 h to 5 μM NP and PCB-77 at 0.001, 0.01 and 1 μM, singly and in combination**. Messenger ribonucleic acid (mRNA) levels were quantified using quantitative (real-time) PCR with gene specific primer pairs. The data are given as % of the solvent control ± standard error of the mean (n = 3). Different letters denote exposure group means that are significantly different for the respective mRNA expression using ANOVA followed by Tukey's multiple comparison test (*p *< 0.05).

Exposure to NP alone significantly elevated ERα expression (Fig. 1A). The low PCB-77 concentration (0.001 μM) produced a significant 2-fold decrease of ERα, compared to control and thereafter a concentration-specific increase of ERα mRNA expression was observed (Fig. 1A). When 1 μM PCB-77 was given in combination with NP, an elevated ERα expression above NP level was observed (Fig. 1A). In contrast, exposure to 0.01 μM PCB-77 in combination with NP produced decreased ERα mRNA below NP level (Fig. 1A). For ERβ, exposure to NP alone produced a significant increase of transcript level (Fig. 1B). When hepatocytes were exposed to 1 μM PCB-77 alone or in combination with NP, ERβ mRNA was not altered (Fig. 1B). In contrast, exposure to 0.001 and 0.01 μM PCB-77 alone produced significant increase of ERβ, and when these PCB-77 concentrations were given in combination with NP, ERβ mRNA was significantly decreased only in the 0.01 μM PCB-77 group (Fig. 1B).

The expression pattern of Vtg was induced 19-fold after exposure to NP alone (Fig. 1C). While PCB-77 alone did not alter the expression levels of Vtg mRNA, the combined exposure with NP produced a PCB-77 concentration-specific decrease of NP induced Vtg expression (Fig. 1C). Particularly, exposure of hepatocytes to NP in combination with medium PCB-77 concentration (0.01 μM) produced a total inhibition of Vtg mRNA expression (Fig. 1C). The expression *Zr*-protein showed a similar pattern with Vtg (Fig. 1D). While exposure to NP alone produced a 3.7-fold increase of *Zr*-protein mRNA, the combined exposure with PCB-77 exposure produced significant PCB-77 concentration-specific decrease of *Zr*-protein, compared with NP exposure alone (Fig. 1D). PCB-77 exposure alone produced significant decrease of *Zr*-protein mRNA expression, compared with solvent control (Fig. 1D). Exposure to PCB-77 concentrations singly or in combination with NP produced minor changes, albeit not significant in vigilin mRNA expression (Fig. 1E). Exposure to PCB-77 concentrations singly or in combination with NP produced non-significant changes in proteasome mRNA expression (Fig. 1F).

### Concentration-dependent expression of AhR-responsive genes

**Figure 2:**
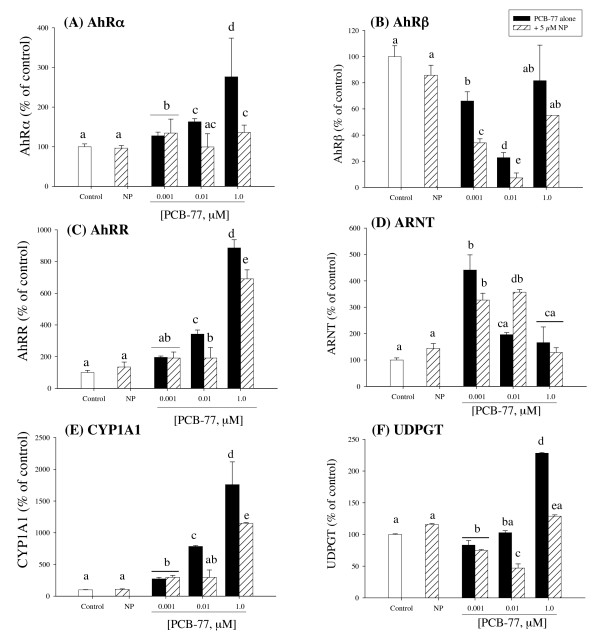
**Expression of AhRα (A), AhRβ (B), AhRR (C), ARNT (D), CYP1A1 (E) and UDPGT (F) mRNA in primary culture of salmon hepatocytes exposed for 48 h to 5 μM NP and PCB-77 at 0.001, 0.01 and 1 μM, singly and in combination**. Messenger ribonucleic acid (mRNA) levels were quantified using quantitative (real-time) PCR with gene specific primer pairs. The data are given as % of the solvent control ± standard error of the mean (n = 3). Different letters denote exposure group means that are significantly different for the respective mRNA expression using ANOVA followed by Tukey's multiple comparison test (*p *< 0.05).

Exposure of hepatocytes to PCB-77 alone produced a significant concentration-dependent increase of AhRα mRNA. While NP alone did not alter AhRα expression, combined NP and PCB-77 at 0.01 and 1 μM caused decreases of AhRα mRNA, compared with PCB-77 exposure alone (Fig. 2A). The expression of AhRβ was significantly decreased after exposure to PCB-77 alone, compared with control (Fig. 2B). Exposure to combined NP and all PCB-77 concentrations showed decreased expression of AhRβ mRNA, significant in 0.001 and 0.01 μM PCB-77 concentrations, compared to PCB-77 exposure alone (Fig. 2B). For AhRR, exposure to PCB-77 alone produced a concentration-dependent increase of AhRR mRNA expression and the presence of NP caused only slight decreases of PCB-77 mediated effects on AhRR expression (Fig. 2C). NP exposure alone did not significantly alter the expression of AhRR mRNA (Fig. 2C). A different expression pattern was observed for ARNT (Fig. 2D). Exposure to the low PCB-77 concentration (0.001 μM) produced a 4.2-fold increase of ARNT mRNA expression and thereafter a PCB-77 concentration-dependent decrease was observed (Fig. 2D). While NP exposure alone produced a slight, albeit not significant, elevation of ARNT mRNA, combined exposure with 0.001 and 0.01 μM PCB-77 produced respective significant decrease and increase of ARNT mRNA expression, compared with the respective PCB-77 concentration alone (Fig. 2D).

The expression pattern of CYP1A1 showed significant PCB-77 concentration-dependent induction and combined exposure with NP produced significant reduction of CYP1A1 mRNA expression, compared with PCB-77 exposure alone (except with 0.001 μM PCB-77; Fig. 2E). NP exposure alone did not alter CYP1A1 mRNA expression (Fig. 2E). Exposure to PCB-77 produced a concentration-specific increase and combined exposure with NP produced significant reduction of UDPGT mRNA expression, compared with PCB-77 exposure alone (except with 0.001 μM PCB-77; Fig. 2F). NP exposure alone did not significantly alter UDPGT mRNA expression (Fig. 2F).

### Time-dependent expression of ER-responsive genes

**Figure 3:**
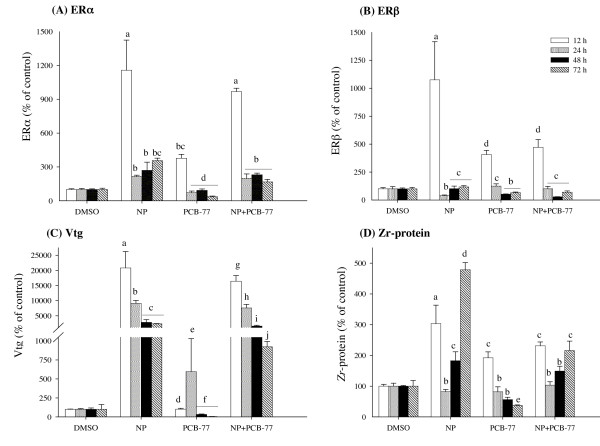
**Time-dependent expression patterns of ERα (A), ERβ (B), Vtg (C) and *Zr*-protein (D) mRNA in primary culture of salmon hepatocytes exposed to 5 μM NP and 1 μM PCB-77, both singly and in combination**. Hepatocytes were sampled at 12, 24, 48 and 72 hours post-aexposure. Expression of mRNA levels was quantified using quantitative (real-time) PCR with gene specific primer pairs. The data are given as % of the solvent control ± standard error of the mean (n = 3). Different letters denote exposure group means that are significantly different for the respective mRNA expression using ANOVA followed by Tukey's multiple comparison test (*p *< 0.05).

Exposure of hepatocytes to NP alone or in combination with PCB-77 caused an apparent time-dependent increase of ERα mRNA expression (Fig. 3A). At 12 h post-exposure, NP exposure singly produced a significant (11-fold) increase of ERα, while combined exposure with PCB-77 slightly reduced (albeit not significant) the NP effect on ERα at the same time interval (Fig. 3A). Although the expression ERα was reduced at 72 h, compared to 12 h, in the NP exposure group alone, the combined exposure with PCB-77 produced significant 2-fold reduction of ERα, compared with NP exposure alone at the same time interval (Fig. 3A). When hepatocytes were exposed to PCB-77 alone, a 3.5-fold increase of ERα mRNA expression was observed at 12 h, and thereafter the expression was reduced below control levels at 24, 48 and 72 post-exposure (Fig. 3A). The expression of ERβ mRNA followed a similar pattern with ERα, but with higher PCB-77 effect (Fig. 3B). Exposure to NP alone produced a significant 11-fold increase of ERβ at 12 h post-exposure and combined NP and PCB-77 exposure resulted to 6-fold reduction compared with NP exposure alone at the same time interval (Fig. 3B). When PCB-77 was given alone, a 4.5-fold increase of ERβ mRNA expression was observed at 12 h after exposure (Fig. 3A). Otherwise, exposure to NP and PCB-77 singly or combined caused minor but variable effects on ERβ mRNA levels at 24, 48 and 72 h after exposure (Fig. 3B).

The expression of Vtg was massively induced (20-fold) after exposure to NP alone at 12 h post-exposure, compared with solvent control (Fig. 3C). Thereafter, Vtg expression in NP-exposed cells showed a time-dependent decreasing trend, albeit massively induced compared to control, at 24, 48 and 72 h after exposure (Fig. 3C). PCB-77 alone produced significant increase of Vtg expression at 24 h post-exposure, compared to control (Fig. 3C). When hepatocytes were exposed to NP and PCB-77 in combination, the NP-induced Vtg expression was reduced at all exposure time points (Fig. 3C). The mRNA expression of *Zr*-proteins increased 3-fold in NP exposed hepatocytes at 12 h post-exposure and decreased back to control level at 24 h (Fig. 3D). Thereafter, a time-dependent increase of *Zr*-protein mRNA, peaking at 72 h, was observed in the NP treated group alone (Fig. 3D). PCB-77 caused significant decreases of *Zr*-protein mRNA expression at 12 and 72 h after exposure, compared to NP treated groups alone (Fig. 3D). When PCB-77 was given alone, a 2-fold increase of *Zr*-protein mRNA was observed at 12 h post-exposure, and thereafter a time-specific decrease was observed (Fig. 3D).

### Time-dependent expression of AhR-responsive genes

**Figure 4:**
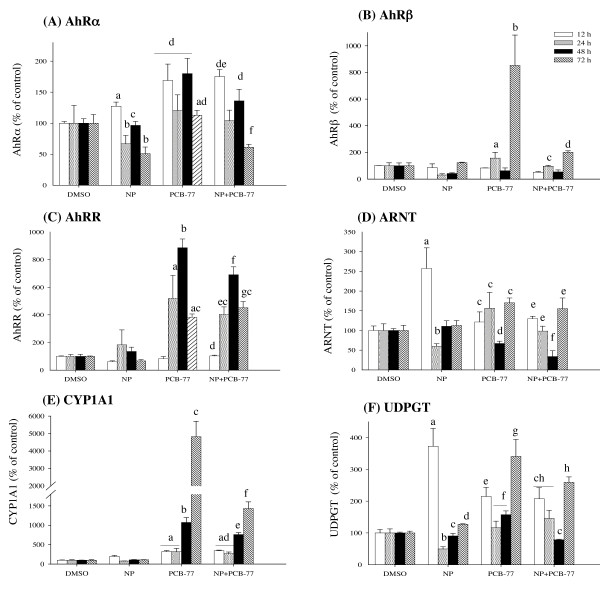
**Time-dependent expression patterns of AhRα (A), AhRβ (B), AhRR (C), ARNT (D), CYP1A1 (E) and UDPGT (F) mRNA in primary culture of salmon hepatocytes exposed to 5 μM NP and 1 μM PCB-77, both singly and in combination**. Hepatocytes were sampled at 12, 24, 48 and 72 hours post-exposure. Expression of mRNA levels was quantified using quantitative (real-time) PCR with gene specific primer pairs. The data are given as % of the solvent control ± standard error of the mean (n = 3). Different letters denote exposure group means that are significantly different, for the respective mRNA expression using ANOVA followed by Tukey's multiple comparison test (*p *< 0.05).

Compared to solvent control, NP caused variable effect on AhRα, producing a 2-fold significant reduction at 72 h post-exposure (Fig. 4A). The AhRα expression increased 2-fold at 12 and 48 h after exposure with PCB-77 alone and combined NP exposure did not produce significant differences, except at 72 h when NP caused 2-fold decrease of PCB-77 induced AhRα expression (Fig. 4A). In contrast, the expression levels of AhRβ mRNA were not significantly affected over time with NP (Fig. 4B). When PCB-77 was given alone, a 2- and 8-fold increase of AhRβ mRNA expression was observed at 24 and 72 h after exposure, respectively (Fig. 4B), while the combined exposure with NP significantly decreased these effects at the corresponding time intervals (Fig. 4B). For AhRR, NP exposure slightly increased the mRNA level at 24 h, but this effect decreased thereafter with time (Fig. 4C). Exposure of hepatocytes to PCB-77 produced a time-specific significant increase of AhRR mRNA expression and these effects were not significantly affected when PCB-77 was given in combination with NP (Fig. 4C). For ARNT, a different pattern of NP-PCB-77 effect was observed (Fig. 4D). NP induced a 2.5-fold significant increase of ARNT at 12 h, and thereafter a 2-fold decrease at 24 h post-exposure was observed, compared to control (Fig. 4D). The ARNT expression in NP exposed group alone returned to control levels at 48 and 72 h post-exposure (Fig. 4D). Exposure to PCB-77 alone produced a 2-fold significant decrease and increase of ARNT mRNA expression at 48 and 72 h, respectively, compared to control (Fig. 4D). When PCB-77 was given in combination with NP, PCB-77 caused respective significant decrease (at 12 and 48 h) and increase (at 24 and 72 h) of NP-mediated ARNT mRNA expression (Fig. 4D). Exposure to PCB-77 singly produced a time-dependent induction of CYP1A1 mRNA reaching 45-fold at 72 h after exposure (Fig. 4E). When hepatocytes were exposed to combined PCB-77 and NP, the PCB-77-induced CYP1A1 mRNA expressions were significantly reduced reaching 15-fold at 72 h post-exposure (Fig. 4E). The UDPGT mRNA expression levels followed a different pattern compared with CYP1A1. NP exposure alone produced a 3.8-fold increase and 1.5-fold decrease of UDPGT expression at 12 and 24 h after exposure, respectively (Fig. 4F). The expression pattern of UDPGT in PCB-77 exposed group alone was generally similar to NP exposure alone, but with non-parallel abundance at 12 and 72 h after exposure. Combined PCB-77 and NP exposure produced decreased UDPGT mRNA expression level at 12 h compared with NP exposure alone. At 72 h, the UDPGT expression was significantly increased in the combined PCB-77 and NP exposure group, compared with NP exposure alone (Fig. 4F).

## Discussion

In the present study, we investigated the ER-AhR interactions and their mediated signalling pathways using agonists for these receptors, genomic methods and *in vitro *system. In our laboratory, we have previously reported that PCB-77, an AhR agonist with known anti-estrogenic activity, caused increases and decreases of *in vivo *ER-mediated NP-induced Vtg and *Zr*-protein gene and protein expression patterns in Atlantic salmon [11]. We found that the *in vivo *responses were dependent on PCB-77 and NP dose ratios and sequential order of exposure and interestingly influenced by seasonal changes [11]. In a recent study, we showed that the partial inhibition of AhR with α-naphthoflavone (ANF) did not reverse the effect of PCB-77 on ER-mediated transcription suggesting that AhRs does not have a direct role on PCB-77 mediated decreases of ER-mediated responses; and the inhibition of ER with tamoxifen (Tam – partial ER antagonist) and ICI 182,780 (ICI – absolute ER antagonist) reversed the transcription of AhR-mediated responses, except AhR repressor (AHRR) [15]. Taken together, these findings demonstrate a complex mode of ER-AhR interaction that is dependent on time- and the individual chemical (NP and PCB-77) concentrations. In order to further characterize the molecular mechanism(s) behind these responses, the analytical power of quantitative (real-time) PCR and salmon primary hepatocyte culture was used with one concentration of NP (5 μM) and different concentrations of PCB-77 (0.001, 0.01 and 1 μM) to study the time-dependent expression patterns of relevant genes in the ER and AhR signalling pathways. Our data show a bi-directional ER-AhR interaction that is dependent on time and PCB-77 concentration.

### Modulation of ER responsive genes

The biological effects of estrogens and their mimics, such as NP are mediated through the ERs. At present, three ER subtypes have been isolated in teleosts. The mRNA transcription of ERα and ERβ, and three estrogen responsive genes (Vtg, *Zr*-protein and vigilin) were studied using real-time PCR. We found that exposure of hepatocytes to NP and PCB-77 singly or in combination produced distinct expression patterns of each ER subtypes, albeit less than NP induced levels. Both ER subtypes (α and β) were significantly altered by NP exposure singly. In mammals, the tissue and cell specific roles of ER isotypes have been described [16]. Tight relationship between the ERα gene isoform expression and Vtg synthesis in a number of teleost species have been reported and strongly suggest that this particular ER plays the dominant role in regulating vitellogenesis [17-19]. In this study, PCB-77 was anti-estrogenic on NP induced Vtg and *Zr*-protein expression in a time-specific manner and these effect showed a parallel pattern of expression with ERα gene expression [20].

### Modulation of AhR responsive genes

We investigated the effects of NP on PCB-77-induced AhR signalling. It should be noted that in this study AhRα and AhRβ are used synonymously with AhR1 and AhR2, respectively. We observed that PCB-77 produced effects on AhR signalling by transcriptional changes of AhR-subtypes (AhRα and AhRβ), ARNT, AhRR, CYP1A1, UDPGT and 20S proteasome subunit. The effects on AhR signalling pathway were dependent on time of exposure and PCB-77 concentration, and were negatively affected by NP. In accordance with the present study, the induced transcription of phase I and II biotransformation enzymes by PCB-77 has previously been reported [9]. The expression of AhRα and AhRR followed a parallel pattern with CYP1A1 and UDPGT after exposure to PCB-77 concentrations. On the contrary, AhRβ and the AhR nuclear dimerization partner, ARNT were differentially affected. For ARNT expression, we observed that a decreased expression pattern with increasing PCB-77 concentration. The overall function of ARNT is not fully understood in teleost, while in mammalian cells, this protein appears to be constitutively active [2]. Although the biochemical and molecular properties of AhR has been characterized in mammalian cells, there are still uncertainties concerning the regulation, interactions with other proteins and transcriptional properties of AhRs [21]. In zebrafish (*Danio rerio*) embryo and liver cell line, TCDD induced a dose-dependent increase of AhR2 mRNA expression [22]. Similar effect was also observed in rainbow trout where the AhR2 and AhR2β were elevated in gonadal cell line and kidney tissue [21]. In addition, these authors did not observe increases in mRNA expression of either AhR2 or AhR2β mRNA after TCDD exposure in rainbow trout liver or spleen [23]. Elsewhere, TCDD or PCB-77 doses did not affect transcriptional changes of AhR2 mRNA expression in Atlantic tomcod (*Microgadus tomcod*) liver [24].

As a transcription factor, the normal physiological and toxicological significance of the multiple AhRs and their associated proteins in many fish species is yet to be fully characterized. In view of the present study and others [25], a comparison of the *in vivo *endogenous response with *in vitro *reporter assays that have utilized different AhR subtypes from rainbow trout suggests that AhRα may account for the CYP1A1 induction by PCB-77 in our system [21]. It has been shown that the amino acid sequence of AhR1 is most closely related to mammalian AhRs which mediate the molecular response after exposure to halogenated aromatic hydrocarbons [26]. The AhR1 (or AhRα) mRNA is nearly undetectable in many tissues that exhibit TCDD (and related compounds)-inducible CYP1A1 expression, implying that AhR2 (or AhRβ) is capable of mediating this response [25]. The transcriptional capability of bHLH-PAS family of transcription factors is yet to be fully understood and their individual *in vivo *functions are still subject of current discussions.

### ER-AhR interactions

Several reports have shown that AhR ligands possess anti-estrogenic properties [11,27,28]. A direct *in vitro *ligand specific interaction between AhR and ERα has been reported by Klinge and co-workers [29]. In our laboratory, a bi-directional ER-AhR interaction has been reported in rainbow trout *in vitro *system [9]. Herein, we show that PCB-77 decreased the expression of NP-induced transcription of ERα, Vtg and *Zr*-protein in a concentration- and time-specific manner. Interestingly, PCB-77 alone significantly increased ERβ expression. Studies of TCDD ability to bind to ER demonstrated that this strong AhR agonist did not compete with E2 for binding to the ER [30]. Four possible mechanisms have been suggested for the anti-estrogenic actions of AhR agonists: 1) increased rate of E2 metabolism; 2) decreased cellular ER isoform levels; 3) suppression of E2 induced transcription; and 4) ER-AhR competition for transcriptional co-factors [31]. Recently, a new mechanism of action termed "ER-hijacking" that defies the above named mechanisms has been postulated [32]. ER-hijacking describes the ability of AhR ligands to activate ER-regulated transcription independent of ER-ligands and has raised the possibility that several xenoestrogens may indeed have estrogenic properties through activation of AhR-ER complex [33]. In fish, we first reported this alternative mode of action for AhR agonists, using PCB-77 and salmon *in vivo *system in 2001 [11]. In that report, we proposed that although the mechanisms by which AhR-agonists induce CYP1A and mediate their antiestrogenic effects seem to be well understood, it could be argued that these mechanisms may be the exception (with regard to estrogen mimics) rather than the rule for the actions of TCDD and related compounds there seem to be ER isoform preferences that favour the α-isoform. Today, several reports have demonstrated that AhR agonists directly induce estrogenic activity through AhR-ERα interactions [[33-35]; Mortensen and Arukwe, in prep]. However, there seem to be ER isoform preferences that favour the α-isoform. For example, a human variant of ERα(-) Ishikawa endometrial cell line were unresponsive to E2, despite their expression of ERβ, reflecting the low transcriptional activity of ERβ compared to ERα [32,33]. Herein, high PCB-77 concentration produced an increase of ERα (also at 12 h post-exposure), above control and statistically equal to NP levels, and in combination with NP produced elevated ERα above NP and control levels. PCB-77 produced an increase of ERβ that was concentration specific, it is possible that AhR agonists, such as PCB-77 may "hijack" both ER subtypes that does not result in the activation of Vtg (but *Zr*-protein at 12 h) response.

When these potential mechanisms are put into context of the present study, degradation of endogenous E2 (or NP) by metabolizing enzymes induced by AhR may lead to decreased ER-mediated transcription. The involvement of CYP1A1 in E2 metabolism was previously investigated in female carp by Smeets and co-workers [36] and reported that the anti-estrogenicity of different AhR ligands in female carp was found to be mediated through the AhR, not involving the CYP1A1. This is in accordance with the present study, showing no clear pattern of decreased ER, Vtg or *Zr*-protein gene expression in response to increased CYP1A1 gene or enzyme activity (measured as 7-ethoxyresorifin O-deethylase, EROD- data not shown) after treatment with PCB-77.

The ER degradation by proteasomes induced by AhR has been explained as another possible anti-estrogenic mechanism [37,38]. In addition to activating AhR, TCDD is found to rapidly reduce the level of AhR protein in cells and mechanistic studies have established that the turnover is mediated through the 26S proteasome, involving ubiquitination of AhR and requires the transcription activation domain of AhR [39,40]. Our data does not support these speculations since despite being expressed there is no direct relationship between a 20S proteasome β-subunit quantified in this study with ERα expression levels. On the contrary, a partial relationship was observed between the proteasome subunit and AhR subtypes, AhRR, CYP1A1 and UDPGT in the combined NP and PCB-77 at 0.01 and 1 μM concentrations. This discrepancy might be caused by the possibility that we may have quantified the wrong proteasome subunit. The choice of proteasome in the present study was based on its differential expression pattern on our subtractive cDNA library after exposure to ER- and AhR-agonists [15]. Furthermore, while the proteasome hypothesis provided us with a rationale for measuring the proteasome gene expression, it should be noted that changes in gene expression are generally not a surrogate for changes in protein degradation due to proteasome degradation. Thus, the proteasome hypothesis should be studied at the protein level.

Previous report have shown that mouse hepatic cell line lacking functional AhR due to mutations in the ARNT, lost ER trans-activation potential in the presence of TCDD due to a sharp decrease in its ability to bind to an ERE [41]. Elsewhere, TCDD prevented reporter gene expression in Xenopus Vtg A2 regulatory sequences even when cells were transiently over-expressing ER, suggesting that the mechanism does not involve ER down-regulation by TCDD [42]. While treatment with E2 increased ER-ERE complex formation, TCDD alone did not have an effect and the binding of ER to ERE was completely lost in cells simultaneously treated with both E2 and TCDD. These observations led the authors to conclude that TCDD was no longer anti-estrogenic in the mutated cell line since AhR was required for the ability of ER to trans-activate from the ERE [41]. When these finding are compared to the data in the present study where PCB-77 produced an apparent concentration-specific increased and decrease of ERα and ARNT, respectively, it is plausible to suggest that PCB-77 mediated anti-NP effect does not involve the down-regulation of ERα expression.

Another possible target for AhR-mediated anti-estrogenicity is the mRNA stability of ER and its transcriptional downstream products (Vtg and *Zr*-proteins). RNA gel mobility shift assays has shown that an estrogen-inducible mRNA stabilizing protein that bound specifically to Vtg mRNA in an area previously implicated in estrogen-mediated stabilization of Vtg mRNA [43]. The stability of mRNA is determined by site-specific mRNA endonuclease activities [44]. The endonuclease catalyzed mRNA decay is regulated through the binding of RNA-binding proteins to target mRNAs that prevent their cleavage by endonucleases [45]. Vigilin, or high density lipoprotein-binding protein, is an ubiquitous protein in vertebrate cells [43]. For example, the stability of liver Vtg mRNA *in Xenopus laevis *is regulated by an E2-induced vigilin that binds specifically to a 3'-untranslated region (3'-UTR) segment of the Vtg mRNA and protects it from degradation [43]. In the present study, the expression of vigilin mRNA in NP exposure singly or in combination with PCB-77 concentration did not produce parallel expression pattern with Vtg or *Zr*-protein. Interestingly, the low PCB-77 exposure alone or in combination with NP that produced an almost total inhibition of Vtg and *Zr*-protein levels showed the highest vigilin expression. We are performing further studies to explain this discrepancy. However, it should be noted that 0.01 μM PCB-77 produced a consistent, but complicated pattern of effect in both ER and some AhR mediated responses (see Figs. 1, 2).

On the AhR signalling pathway, we observed that the NP decreased the transcription of AhRα, AhRβ, AhRR, ARNT, CYP1A1 and UDPGT to below PCB-77 exposed levels in a PCB-77 concentration- and time-specific manner, indicating that NP has anti-AhR signalling effects. Interestingly, the expression of AhRβ and ARNT showed a different pattern of effect in PCB-77 exposure alone and in combination with NP. We observed PCB-77 exposure first induced ARNT at low concentration and thereafter a concentration-specific decrease was observed. ARNT functions as a dimerization partner for several proteins in the bHLH-PAS protein superfamily [2,28], therefore, only minor alterations in ARNT gene expression could be expected in response to xenobiotic exposures. However, on the basis of sequence homology with an ER transcription factors p160, it was shown that ARNT functions as a co-activator of ER and this effect was due to the C-terminal domain and not the conserved bHLH or PAS domains [28]. In addition, although the ARNT contains a less complex activation domain compared to AhR; the activation domains of AhR and ARNT are located in the carboxy-terminal of both genes [46]. During CYP1A1 (and other genes) activation, the ARNT activation domain does not contribute to the activation of AhR complex [47].

In general, the present data are consistent with previous studies showing that NP (*i.e.*, estrogen mimic) and E2 significantly suppressed hepatic CYP1A1 mRNA levels, EROD activity and CYP1A1 protein in *in vivo *and *in vitro *experiments using several teleost species [48,49]. Based on the possible mechanisms explained above, we hypothesize that NP can bind the CYP1A1 protein [50], and through this binding, NP or its metabolites may inhibit the CYP1A1 expression [51]. Alternatively, the effect of NP could partially be mediated by the liver ERs through a process that may involve the ER-NP complex interfering with the AhR transcription machinery either directly or with the CYP1A1, or indirectly through bind to the XRE and regulating AhR-induced gene expression. In addition, NP may control the recruitment of ER and possibly other co-activators, besides activating the detoxification pathway.

The consistency between AhRR, CYP1A1 and UDPGT expression pattern suggests that this repressor singly may have caused the decrease in CYP1A1 and UDPGT levels. The AhRR-ARNT heterodimerization may negatively regulate AhR driven gene expression through transcriptional repression [52]. In accordance with our data, the modulation of CYP1A1 by NP, E2, and BNF was recently shown to parallel the AhRR gene expression [53]. Any of the above mentioned mechanisms might have caused the NP effect on AhR signalling. This is supported by the fact that the BHLH-PAS (Per-AhR/ARNT-Sim homology sequence) of transcription factor usually associate with each other to form heterodimers, AhR/ARNT or AhRR/ARNT, and bind the XRE sequences in the promoter regions of the target genes to regulate their expression.

## Conclusion

The findings in the present study demonstrate the interactions between NP and PCB-77 in primary culture of salmon hepatocytes. The AhR-agonist (PCB-77) functioned as anti-NP-mediated effect, and NP functioned as anti-AhR-mediated effect or as inhibitor of AhRα, AhRR, ARNT, CYP1A1 and UDPGT expression. Overall, the findings demonstrate a complex mode of ER-AhR interactions that were dependent on the time of exposure and individual chemical (NP and PCB-77) concentrations. A novel aspect of the present study is that low (0.001 μM) and medium (0.01 μM) PCB-77 concentrations increased ERβ mRNA expression above control and NP levels, and at 12 h post-exposure, PCB-77 exposure alone produced significant elevation of ERα, ERβ and *Zr*-protein expressions above control levels. Nevertheless, a retrospective evaluation of the data presented here showed that 12 h could have been a better exposure time for the concentration study since it was at this time point most unique responses were observed. However, the choice of our exposure time was based on previous studies in our laboratory (and elsewhere) that have produced significant interactions between NP and PCB-77 is fish primary hepatocyte culture. In our laboratory, we are still performing studies on cross-talk between the ER-AhR signal transduction systems and underlying mechanism(s) by which xenobiotics and xenoestrogens interact with each other. This complex interaction between two different classes of ligand-activated receptors provides novel mechanistic insights on signalling pathways.

## Methods

### Chemicals and reagents

4-nonylphenol (NP; 85% of p-isomers) was purchased from Fluka Chemika-Biochemika (Buchs, Switzerland). The impurities in 4-nonylphenol consist mainly of phenol (8–13%), tripropylene (~1%) and 2,4-dinonylphenol (~1%). 3,3',4,4'-Tetrachlorobiphenyl (PCB-77; 99.7% pure) was purchased from Dr. Ehrenstorfer GmbH (Augsburg, Germany). Dulbecco minimum essential medium (DMEM) with non-essential amino acid and without phenol red, fetal bovine serum (FBS), L-glutamine and TA cloning kit were purchased from Gibco-Invitrogen Life Technologies (Carlsbad, CA, USA). Dimethyl sulfoxide (DMSO), 100× penicillin-streptomycin-neomycin solution, collagenase, bovine serum albumin (BSA), N-[2-hydroxyethyl]piperazine-N'-[2-ethanesulfonic acid] (HEPES), ethyleneglycol-bis-(β-aminoethylether) N, N'tetraacetic acid, (EGTA), 0.4% trypan blue were purchased from Sigma Chemical (St. Louis, MO, USA). E.Z.N.A. total RNA kit for ribonucleic acid (RNA) purification was from Omega Bio-Tek (Doraville, GA, USA). IScript cDNA synthesis kit and iTAQ™ SYBR^® ^green supermix with ROX were purchased from Bio-rad Laboratories (Hercules, CA, USA). GeneRuler™ 100 base pairs (bp) DNA ladder and deoxynucleotide triphosphates (dNTPs) were purchased from Fermentas GmbH (St. Leon-Rot, Germany).

### Collagenase perfusion, isolation and culture of hepatocytes

Juvenile Atlantic salmon (*Salmo salar*) of approximately 400–500 g were supplied by Marine Harvest AS, Dyrvik, Norway and kept at the animal holding facilities at the Biology Department, NTNU. Fish were supplied with continuously running saltwater at a constant temperature of 10°C. Prior to liver perfusion all glassware and instruments were autoclaved before use. Solutions were filtration sterilized by using 0.22 μm Millipore filter (Millipore AS, Oslo, Norway). Hepatocytes were isolated from 3 individuals (triplicate exposures) by a two-step perfusion technique with modifications as described by Andersson and co-workers [54]. The cell suspension was filtered through a 150 μm nylon monofilament filter and centrifuged at 50 × g for 5 min. Cells were washed three times with serum-free medium and finally resuspended in complete medium. Following collagenase perfusion and isolation of hepatocytes, viability of cells was determined by the trypan blue exclusion method. A cell viability value of > 90% was a criterion for further use of the cells. Cells were plated on a 35 mm Primaria culture plates (Becton Dickinson Labware, USA) at the recommended density for monolayer cells of 5 × 10^6 ^cells in 3 ml DMEM medium (without phenol red) containing 2.5% (v/v) FBS, 0.3 g/L glutamine, and 1% (v/v) penicillin-streptomycin-neomycin solution. The cells were cultured at 10°C in a sterile incubator without additional O_2_/CO_2 _for 48 hr prior to chemical exposure.

### Exposure of hepatocytes

After 48 h pre-culture, two separate experiments were performed. Firstly, we evaluated the effects of different PCB-77 concentrations on NP mediated effects. Secondly, we investigated the time-response pattern of these effects. Both NP and PCB-77 concentrations were chosen based on previous experiments. These studies showed that these concentrations are optimal *in vitro *concentrations for ER-AhR interactions in salmonids [9]; Mortensen and Arukwe, submitted). In the first experiment, hepatocytes were exposed (triplicate plates for each exposure group) for 48 h to 0.01% DMSO (control), 5 μM NP and 0.001, 0.01 and 1 μM PCB-77 singly and also in combination. In the second experiment, hepatocytes were exposed (triplicate plates for each exposure group) for 12, 24, 48 and 72 h to 0.01% DMSO (control), 5 μM NP and 1 μM PCB-77 singly and also in combination. In both experiments, media were replaced with fresh media containing the respective test chemical and concentrations every 24 h. Media and cells were harvested after exposure and lysed in E.Z.N.A lysis buffer for total RNA isolation according manufacturers protocol (Omega Bio-Tek).

### Quantitative (real-time) PCR

Total cDNA for the real-time PCR reactions were generated from 1 μg total DNase-treated RNA from all samples using poly-T primers from iScript cDNA Synthesis Kit as described by the manufacturer (Bio-Rad). Quantitative (real-time) PCR was used for evaluating gene expression profiles. For each treatment, the expression of individual gene targets was analyzed using the Mx3000P REAL-TIME PCR SYSTEM (Stratagene, La Jolla, CA, USA). Each 25-μL DNA amplification reaction contained 12.5-μL of iTAQ™ SYBR^® ^Green Supermix with ROX (Bio-Rad), 1 μL of cDNA and 200 nM of each forward and reverse primers. The 3 step real-time PCR program included an enzyme activation step at 95°C (5 min) and 40 cycles of 95°C (30 sec), 55–65°C for 30 sec, depending on the primers used (see Table 1), and 72°C (30 sec). Controls lacking cDNA template (minus reverse transcriptase sample) were included to determine the specificity of target cDNA amplification as described previously [9,55]. Briefly, cycle threshold (Ct) values obtained were converted into mRNA copy number using standard plots of Ct versus log copy number. The criterion for using the standard curve is based on equal amplification efficiency with unknown samples and this is usually checked prior to extrapolating unknown samples to the standard curve. The standard plots were generated for each target sequence using known amounts of plasmid containing the amplicon of interest. Data obtained from triplicate runs for target cDNA amplification were averaged and expressed as ng/μg of initial total RNA used for reverse transcriptase (cDNA) reaction. Standard errors were calculated using S-plus statistic software 6.2 (Insightful Corp, USA). Statistical differences among treatment groups were tested using analysis of variance (ANOVA) and comparison of different exposure treated and control groups were performed using Tukey's multiple comparison test. The multiparametric ANOVA test was performed after testing for normality and also variance homogeneity, using the Levene's test. For all the tests the level of significance was set at *p *< 0.05, unless otherwise stated.

**Table 1:** Primer pair sequences, accession numbers, amplicon size and annealing temperature conditions for genes of interest used for real-time PCR.

**Target Gene**	Primer sequence*		**Amplicon size (nucleotides)**	**Annealing temperature (°C)**	**GenBank accession number**
				
	Forward	Reverse			
ERα	TCCAGGAGCTGTCTCTCCAT	GATCTCAGCCATACCCTCCA	173	55	DQ009007
ERβ	GAGCATCCAAGGTCACAATG	CACTTTGTCATGCCCACTTC	126	59	AY508959
Vtg	AAGCCACCTCCAATGTCATC	GGGAGTCTGTCCCAAGACAA	391	57	DY802177
*Zr*-protein	TGACGAAGGTCCTCAGGG	AGGGTTTGGGGTTGTGGT	113	55	AF407574
Vigilin	GGGATACGCACAGACACCTT	CCCAGATTCCACAGACACCT	86	60	DY802195
AhRα	AGGGGCGTCTGAAGTTCC	GTGAACAGGCCCAACCTG	82	60	AY219864
AhRβ	GACCCCCAGGACCAGAGT	GTTGTCCTGGATGACGGC	96	65	AY219865
AhRR	TTCCTCCAGGGACAGAAGAA	ATGGAGGGCAGCAGAAGAG	98	60	DQ372978
Arnt	AGAGCAATCCCAGGGTCC	TGGGAGGGTGATTGAGGA	107	60	DQ367887
CYP1A1	GAGTTTGGGCAGGTGGTG	TGGTGCGGTTTGGTAGGT	76	60	AF364076
UDPGT	ATAAGGACCGTCCCATCGAG	ATCCAGTTGAGGTCGTGAGC	113	55	DY802180
Proteasome	TCTTTGACCAGGTTGCACAG	CATACAAAGCTGGTGGCTCA	134	60	DY802110

## Competing interests

The author(s) declare that they have no competing interests.

## Authors' contributions

ASM carried out the experiments, processed the data and participated in writing the manuscript. AA initiated the study, designed and supervised the study. All authors read and approved the final manuscript
